# Case Report: Niraparib–abiraterone in HRR-mutated metastatic castration-resistant prostate cancer: clinical insights from five cases

**DOI:** 10.3389/fonc.2026.1799304

**Published:** 2026-04-27

**Authors:** Ismaela Anna Vascotto, Martina Catalano, Jacopo Venturini, Chiara Calandrelli, Silvia Mancini, Luca Pratesi, Martina Izzi, Marinella Micol Mela, Virginia Rossi, Edoardo Francini, Serena Pillozzi, Lorenzo Antonuzzo, Giandomenico Roviello

**Affiliations:** 1 Department of Experimental and Clinical Medicine, University of Florence, Florence, Italy; 2 Department of Health Sciences, Section of Clinical Pharmacology and Oncology, University of Florence, Florence, Italy; 3 Clinical Oncology, Careggi University Hospital, Florence, Italy

**Keywords:** abiraterone acetate, BRCA, mCRPC, niraparib, PSA

## Abstract

**Background:**

Combination therapy with the PARP inhibitor niraparib and the androgen-receptor inhibitor abiraterone acetate plus prednisone (AAP) has recently shown improvement in radiographic progression-free survival (rPFS) in patients with metastatic castration-resistant prostate cancer (mCRPC) harboring homologous recombination repair (HRR) gene alterations, particularly BRCA1/2, in the phase III MAGNITUDE trial. Evidence outside clinical trials remains limited.

**Methods:**

We retrospectively reviewed the clinical courses of five consecutive patients with HRR-mutated mCRPC treated with niraparib (200 mg once daily) plus abiraterone acetate (1000 mg once daily) and prednisone (5 mg twice daily) at our institution. Baseline characteristics, PSA kinetics (including time to PSA 50% decline), radiological responses, progression-free survival (PFS; defined as a composite clinical, radiological, and biochemical endpoint), overall survival (OS), and safety outcomes were assessed. All patients underwent radiological evaluation every three months, including contrast-enhanced CT of the chest and abdomen and bone scintigraphy. Tumor response was assessed according to RECIST 1.1 criteria for measurable disease.

**Results:**

All patients harbored pathogenic HRR gene alterations: somatic and/or germline BRCA1/2 (three BRCA2, two BRCA1), and one patient with concurrent PALB2. Median age at therapy initiation was 70 years (range 60–80). Three patients (Patients 1, 4, 5) achieved significant and sustained PSA declines (≥50%) and prolonged disease control, including elderly and frail individuals. Two patients (Patients 2, 3) exhibited early progression with limited clinical benefit, consistent with aggressive disease course. Median PFS varied widely across the cohort, reflecting heterogeneity of clinical phenotypes. Treatment was generally manageable; dose interruptions or reductions were required in selected cases, with no new safety signals observed.

**Conclusions:**

Niraparib–abiraterone demonstrated anti-tumor activity in a subset of HRR-mutated mCRPC patients, including elderly and frail individuals. However, the very small sample size, retrospective design, and absence of standardized rPFS assessment limit generalizability. These findings are primarily hypothesis-generating but align with prospective trial results and underscore the heterogeneity of HRR-mutated mCRPC, highlighting the need for individualized therapeutic strategies.

## Introduction

Metastatic castration-resistant prostate cancer (mCRPC) remains a significant clinical challenge, particularly in patients harboring defects in homologous recombination repair (HRR) genes. Approximately 20–25% of men with mCRPC carry germline or somatic mutations in DNA repair genes, including BRCA1/2 and ATM, conferring increased sensitivity to PARP inhibition (1, 2). Preclinical studies have demonstrated that inhibition of PARP, a key enzyme in single-strand DNA repair, induces synthetic lethality in tumors with defective homologous recombination, particularly when combined with androgen receptor (AR) pathway suppression (3).

Recent clinical investigations have evaluated the efficacy of combining PARP inhibitors with standard AR-targeted therapies in HRR-mutated mCRPC. The MAGNITUDE trial assessed the combination of niraparib (Akeega) with abiraterone acetate and prednisone in men with HRR-deficient mCRPC. Initial results demonstrated a significant improvement in rPFS compared with standard therapy alone, highlighting the potential of this combination as a novel treatment strategy (4, 5).

However, randomized trials often enroll selected patients with stringent eligibility criteria and limited comorbidities, hindering generalizability. Therefore, small-scale observational studies are valuable to characterize outcomes across different patient populations, including elderly and frail individuals. Here, we report five cases of HRR-mutated mCRPC treated with niraparib–abiraterone, focusing on clinical features, PSA kinetics, radiologic responses, outcomes, and safety.

## Methods

### Study design and patients

This retrospective case series included five consecutive patients with mCRPC treated with niraparib–abiraterone at our institution between February 2024 and April 2025. These five cases represent all HRR-mutated mCRPC patients consecutively treated with niraparib–abiraterone at our institution during the study period, without any selection criteria. All patients had histologically confirmed prostate adenocarcinoma, evidence of castration-resistant disease, and at least one pathogenic HRR gene alteration identified through somatic or germline testing. All patients continued androgen deprivation therapy (ADT) throughout treatment, in accordance with standard clinical practice, and provided informed consent for anonymized analysis of clinical data.

### Data collection and endpoints

Baseline clinical characteristics, prior therapies, PSA values, imaging findings, treatment duration, adverse events (CTCAE v5.0) (6), and survival status were extracted. PSA response was defined as a ≥50% decline from baseline. PSA90 response (≥90% decline from baseline) was also recorded when applicable. PSA halving time was calculated as the time required to achieve a 50% reduction from baseline PSA. Due to the retrospective nature of the study and variability in clinical practice, PSA progression was not uniformly defined according to PCWG3 criteria.

PFS was defined as a composite real-world endpoint, including radiological, clinical, or biochemical progression, or death. Due to the retrospective design and the absence of centralized and standardized radiological assessment, radiographic progression-free survival (rPFS) according to clinical trial criteria could not be formally assessed. OS was time from therapy initiation to death or last follow-up.

### Radiological assessment

Patients underwent contrast-enhanced CT scans of the chest and abdomen and bone scintigraphy every three months. Tumor responses were evaluated according to RECIST 1.1 criteria for measurable disease. Imaging findings were integrated with clinical and biochemical data to document disease progression.

## Results

**Figure 1:**
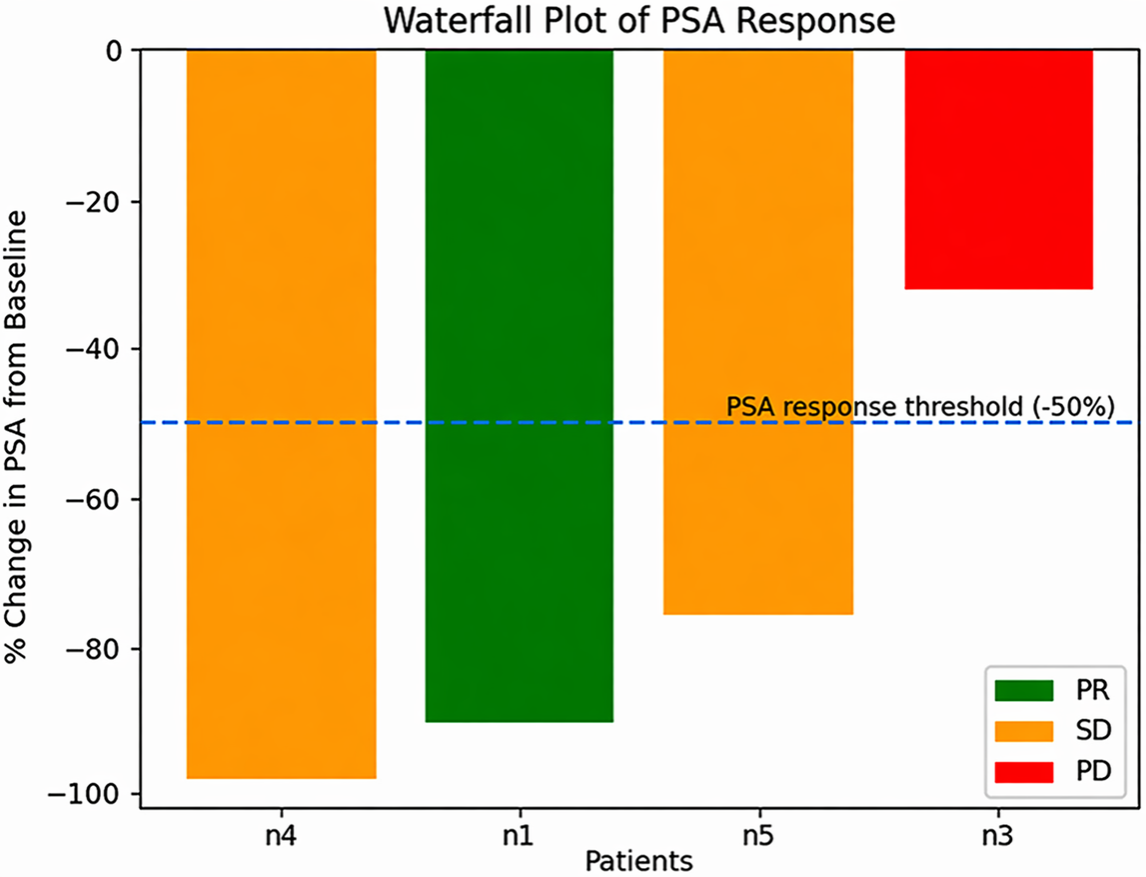
Maximum percentage change in PSA from baseline for each evaluable patient. Bars are color-coded by best radiological response (PR, SD, PD). The dashed line indicates the −50% PSA response threshold.

Baseline clinical and molecular characteristics are summarized in **Table 1**, providing detailed information on performance status, prior treatments, disease burden, and genomic alterations. Median age at Akeega initiation was 70 years (range 60–80). HRR alterations included BRCA2 mutations in three patients, BRCA1 mutations in two patients, and a concurrent PALB2 mutation in one patient. All patients had metastatic disease; four had bone metastases, and three had lymph node involvement. **Figure 1** reported the maximum percentage change in PSA from baseline for each evaluable patient.

**Table 1:** Baseline clinical and molecular characteristics of patients.

Patients	n1	n2	n3	n4	n5
Age (y)	65	78	67	80	60
ECOG PS	0	1	0	1	0
Gleason score	9 (4 + 5)	9 (5 + 4)	9 (5 + 4)	10 (5 + 5)	9 (4 + 5)
Comorbidities	none	Arterial hyp., diabetes mellitus, Dyslipidemia	Genetic Hypoacusis, HCV positive	Dyslipidemia	Arterial hyp., Dyslipidemia
HRR gene	BRCA2	BRCA1	BRCA2	BRCA1	BRCA2 PALB2
Germline/ Somatic	Germline + somatic	Somatic	Somatic	Somatic	Somatic + Germline
Mutation type	c.9026_9030del, p.(Tyr3009SerfsTer7)	c.1016del, p.(Lys339ArgfsTer2	c.5351delA, p.(Asn1784fs)	c.4066C>T, p.(Gln1356Ter); c.3329del, p.(Lys1110SerfsTer7)	BRCA2: c.1813delA, p.(Ile605TyrfsTer9 PALB2: c.1592T>A, p.(Leu531Ter)
VAF (%)	NA	1.61	1.27	11.8; 11	3.81
Metastatic at diagnosis (yes/no)	Yes	Yes	Yes	No	Yes
Date of diagnosis	June 2021	July 2023	January 2024	March 2023	May 2023
Metastatic sites	Bone	Bone, Lymph nodes	Bone, Lymph nodes	Bone	Bone, Lymph nodes
N. metastatic lesions (<5/5-10/> 10)	> 10	5-10	> 10	< 10	5-10
Prior systemic therapies	ADT + Docetaxel	ADT + Apalutamide	ADT + Docetaxel	ADT	ADT+ Docetaxel + Darolutamide
Setting prior treatment	mHSPC	mHSPC	mHSPC	HSPC	mHSPC
Baseline PSA (ng/ml)	19	319	6.27	29.47	7.71
Date of PSA baseline	February 2024	October 2024	September 2024	April 2025	February 2025
PSA nadir during Akeega	1.94	NA	4.25	0.54	1.89
Date of PSA nadir	October 2025	NA	NA	December 2025	September 2025
Best response	PR	PD	PD	SD	SD

### Patient 1 (65 years, BRCA2 germline + somatic)

A 65-year-old man with metastatic, high-grade prostate adenocarcinoma (Gleason 4 + 5) and disseminated bone metastases enrolled after progressive disease post-docetaxel exposure. Genetic testing revealed BRCA2 mutation. Baseline PSA at Akeega initiation was 19 ng/mL, rising from 9.2 ng/mL three months prior. After starting niraparib–abiraterone, PSA initially rose to 35 ng/mL at one month, then declined progressively to a nadir of 1.94 ng/mL by October 2025, representing an ~90% reduction from baseline. Radiological imaging demonstrated disease stabilization on contrast-enhanced CT scans and bone scintigraphy, with reduction of metastatic burden and resolution of several osseous lesions. OS and PFS exceeded 18 months at last follow-up. Treatment was well tolerated. This patient demonstrated deep biochemical and radiological response, consistent with clinical benefit from PARP plus AR targeting.

### Patient 2 (78 years, somatic BRCA1)

A 78-year-old male with high-grade (Gleason 5 + 4) mCRPC and extensive lymph node and skeletal involvement received first-line niraparib–abiraterone after progression on apalutamide. Baseline PSA was 319 ng/mL. Disease progressed rapidly with minimal PSA decline and clinical deterioration. The patient discontinued therapy shortly thereafter and succumbed to disease approximately 1.5 months after Akeega initiation. PSA kinetics and imaging findings consistently indicated rapidly progressive disease with no meaningful response to treatment. This case illustrates primary resistance and limited benefit despite BRCA1 mutation, highlighting disease heterogeneity.

### Patient 3 (67 years, somatic BRCA2)

A 67-year-old man with mCRPC after docetaxel and initial hormonal therapy in the mCSPC setting, harboring a low-frequency somatic BRCA2 mutation, started niraparib–abiraterone with a baseline PSA of 6.27 ng/mL. PSA initially decreased modestly then rose, reaching 38.9 ng/mL by 29 weeks, coinciding with radiological progression. A subsequent CT scan of the chest and abdomen revealed widespread metastatic progression. The patient died in July 2025. This case highlights suboptimal response despite BRCA2 alteration. PSA trends showed only a transient and modest decline followed by a rapid increase, in line with radiological progression. Overall, this pattern reflects limited and short-lived clinical benefit.

### Patient 4 (80 years, somatic BRCA1)

An 80-year-old frail patient with high-grade adenocarcinoma (Gleason 5 + 5) and bone metastases was started on niraparib–abiraterone with baseline PSA 29.47 ng/mL. Over time, PSA declined progressively, reaching a nadir of 0.54 ng/mL by December 2025. The treatment course was complicated by the emergence of behavioral changes, necessitating a temporary interruption of dosing. Radiologic assessment demonstrated stable disease. This case underscores that even very elderly and frail patients may derive benefit with careful supportive care.

### Patient 5 (60 years, somatic BRCA2 + PALB2)

A 60-year-old male with *de novo* high-volume disease, characterized by multiple bone and lymph node metastases. The patient harbors a somatic BRCA2 mutation with a concurrent PALB2 co-mutation. He started treatment with niraparib–abiraterone following prior therapy with the triplet regimen docetaxel + darolutamide + leuprorelin (enantone) in the mCSPC setting. Baseline PSA at Akeega initiation was 7.71 ng/mL, with subsequent nadir of 1.89 ng/mL. Radiologic assessments showed disease stabilization with intermittent focal radiotherapy for symptomatic bone lesions. PSA kinetics and imaging supported prolonged disease control; OS and PFS were exceeding 10 months at last evaluation. Hematologic toxicity (anemia, thrombocytopenia grade 2) required temporary therapy suspension.

### Summary of PSA kinetics and outcomes

Three of five patients achieved ≥50% PSA declines, including one patient who achieved a ≥90% PSA reduction, while two exhibited primary resistance. Safety outcomes were consistent with known PARP inhibitor and ARPI toxicities, manageable with dose adjustments or temporary holds.

## Discussion

This five-patient case series illustrates the heterogeneity of responses to niraparib–abiraterone in HRR-mutated mCRPC. The efficacy of this combination has been established in the phase III MAGNITUDE trial, particularly in patients with BRCA1/2 alterations (4, 5). Compared with pivotal trials, our cohort reflects a more heterogeneous and less selected real-world population, including elderly and frail patients who are often underrepresented in clinical trials. Variability in outcomes appeared more pronounced in our series. Three patients achieved profound and sustained PSA responses, reflecting benefits reported in pivotal trials (7, 8). Conversely, two patients exhibited rapid progression despite pathogenic BRCA alterations, highlighting the biological heterogeneity of HRR-altered mCRPC and the potential for intrinsic resistance mechanisms beyond BRCA status alone (8, 9). Real-world evidence on PARP inhibitor combinations in mCRPC remains limited. However, available studies and recent meta-analyses suggest that treatment outcomes may differ from those observed in clinical trials due to broader patient heterogeneity, comorbidity burden, and variability in treatment management (7–9). Emerging evidence indicates that factors such as allelic status, mutation clonality, and the broader co-mutational landscape may critically influence PARP inhibitor sensitivity and shape clinical outcomes (10, 11). In this context, variant allele frequency (VAF), when available, may serve as a surrogate marker of mutation clonality. Low VAF may reflect subclonal alterations, potentially resulting in incomplete homologous recombination deficiency and reduced sensitivity to PARP inhibitors (**Table 1**) (12, 13). In clinical practice, these molecular features may also have implications for treatment sequencing decisions, as patients with subclonal or low-VAF alterations may derive less benefit from PARP inhibitors and could be considered for alternative or earlier therapeutic strategies (12, 13). In our case series, the two patients who experienced rapid disease progression both harbored somatic BRCA1/2 mutations with low VAF, which may have contributed to their limited or short-lived clinical benefit. By contrast, patient 1 carried a germline BRCA2 mutation, a molecular context generally associated with more durable responses. Of particular note, patient 5 harbored a concurrent PALB2 co-mutation in addition to a somatic BRCA2 alteration. While the clinical significance of concurrent HRR alterations remains incompletely defined, such molecular complexity may further modulate PARP inhibitor sensitivity and clinical outcomes. These observations reinforce that HRR-mutated mCRPC represents a biologically diverse entity in which response is likely determined by multiple genomic and clinical factors rather than by BRCA status alone (7–9).

Safety outcomes were consistent with the known toxicity profile of PARP inhibitors combined with androgen receptor pathway inhibitors, including hematologic adverse events and fatigue, and were generally manageable with dose adjustments or temporary treatment holds (4, 5).

Finally, several limitations must be acknowledged. This case series represents the entirety of our institutional experience with this treatment, reducing the likelihood of selection bias, although it remains limited by its small sample size, retrospective design, and single-center nature. Therefore, findings should be considered descriptive and hypothesis-generating rather than definitive. In particular, the lack of standardized radiographic assessment precluded formal evaluation of rPFS, limiting direct comparability with randomized clinical trials. Additionally, PSA-based endpoints were not uniformly assessed according to PCWG3 criteria, reflecting variability in real-world clinical practice and given the very small sample size, Kaplan–Meier survival curves were not performed. Nevertheless, the consistency of selected observations with data from prospective trials supports the biological plausibility of our results and underscores the importance of comprehensive molecular profiling to guide individualized therapeutic strategies.

## Conclusions

In this case series, niraparib–abiraterone demonstrated meaningful PSA responses and durable disease control in selected HRR-mutated mCRPC patients, including elderly and frail individuals. The heterogeneity of clinical outcomes highlights the influence of genomic context, co-mutations, and patient-specific factors. These findings reinforce the value of comprehensive molecular profiling, including the assessment of variant allele frequency when available, to support individualized treatment decisions in advanced prostate cancer.

## Data Availability

Dataset is available on a request. Requests to access these datasets should be directed to giandomenico.roviello@unifi.it.
